# Spatio-Temporal Transmission Patterns of Black-Band Disease in a Coral Community

**DOI:** 10.1371/journal.pone.0004993

**Published:** 2009-04-01

**Authors:** Assaf Zvuloni, Yael Artzy-Randrup, Lewi Stone, Esti Kramarsky-Winter, Roy Barkan, Yossi Loya

**Affiliations:** 1 Department of Zoology, Tel Aviv University, Ramat Aviv, Tel Aviv, Israel; 2 The H. Steinitz Marine Biology Laboratory, the Interuniversity Institute for Marine Sciences of Eilat, Eilat, Israel; 3 Biomathematics Unit, Department of Zoology, Tel Aviv University, Ramat Aviv, Tel Aviv, Israel; 4 Department of Geophysics and Planetary Sciences, Tel-Aviv University, Ramat Aviv, Tel Aviv, Israel; University of North Carolina at Chapel Hill, United States of America

## Abstract

**Background:**

Transmission mechanisms of black-band disease (BBD) in coral reefs are poorly understood, although this disease is considered to be one of the most widespread and destructive coral infectious diseases. The major objective of this study was to assess transmission mechanisms of BBD in the field based on the spatio-temporal patterns of the disease.

**Methodology/Principal Findings:**

3,175 susceptible and infected corals were mapped over an area of 10×10 m in Eilat (northern Gulf of Aqaba, Red Sea) and the distribution of the disease was examined monthly throughout almost two full disease cycles (June 2006–December 2007). Spatial and spatio-temporal analyses were applied to infer the transmission pattern of the disease and to calculate key epidemiological parameters such as 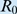 (basic reproduction number). We show that the prevalence of the disease is strongly associated with high water temperature. When water temperatures rise and disease prevalence increases, infected corals exhibit aggregated distributions on small spatial scales of up to 1.9 m. Additionally, newly-infected corals clearly appear in proximity to existing infected corals and in a few cases in direct contact with them. We also present and test a model of water-borne infection, indicating that the likelihood of a susceptible coral becoming infected is defined by its spatial location and by the relative spatial distribution of nearby infected corals found in the site.

**Conclusions/Significance:**

Our results provide evidence that local transmission, but not necessarily by direct contact, is likely to be an important factor in the spread of the disease over the tested spatial scale. In the absence of potential disease vectors with limited mobility (e.g., snails, fireworms) in the studied site, water-borne infection is likely to be a significant transmission mechanism of BBD. Our suggested model of water-borne transmission supports this hypothesis. The spatio-temporal analysis also points out that infected corals surviving a disease season appear to play a major role in the re-introduction of the disease to the coral community in the following season.

## Introduction

There is growing concern over the effects of coral diseases on coral communities throughout the world. During the past two decades the frequency and virulence of coral diseases have increased worldwide [1]–[5], and it has been suggested that recent increases in disease outbreaks may be associated with environmental stressors, including increased seawater temperatures, variation in salinity, pollution, sedimentation and eutrophication [2], [6]–[10]. In scleractinian corals, infectious diseases are recognized as important factors affecting community composition, structure and dynamics [11]. In some coral reef ecosystems, such as in the Western Atlantic, disease outbreaks have appeared as one of the primary causes of the accelerating destruction of the reefs [1]–[2], [12]–[14].

Most of the quantitative information available on the prevalence of coral diseases and their impact on coral populations and communities has been gathered in the Caribbean. However, even there, to date there is only a very limited understanding of the dynamics driving these diseases, so that many fundamental questions remain unresolved. There is a recognized need to protect coral reef communities from regional-scale infections, such as those that led to the mass mortalities in the Caribbean. Marine reserve managers are awaiting the development of remediation and restoration protocols to be included in optimal policy guidelines for effective management programs [4]. However, it is understood that any progress in this direction first requires a far more refined understanding of the key ecological processes controlling the dynamics and spread of coral disease infections.

Black-band disease (BBD) is one of the most widespread and destructive coral infectious diseases [15]. It affects a number of known reef framework-building coral species. A comprehensive list of coral species affected by BBD is presented in Green and Bruckner [16]. BBD commonly exhibits very low prevalence (percentage of corals infected) of less than 1% when active on reefs [17]–[19]. Despite such low occurrences, its persistence makes it an important factor in structuring coral reef ecosystems [20]. In addition, infected corals have not been observed to recover to any great extent, and the newly-exposed substrate (dead coral) remains bare of coral recruits for many years [21]–[22]. BBD was primarily recorded as present on reefs throughout the Caribbean in the 1970s [23]. In the 1980s it was found to also occur in the Indo-Pacific [24] and in the Red Sea [25], and by the 1990s it had spread to the Great Barrier Reef [19], by now exhibiting a global distribution.

Even though BBD was the first coral disease to be studied [23], there are many unresolved questions concerning the mode of transmission of this disease on the reef, including the mechanism by which it has spread to infect corals worldwide. To date, the little information we have regarding the modes of transmission of BBD infections is mainly based on laboratory experiments. Rützler et al. [26] suggested that healthy corals can become infected with BBD by direct contact, and that infections are not seen on healthy coral specimens when placed at a distance as small as 2 mm away from infected corals. However, injured corals have been found to be more susceptible to the disease and become infected with BBD when placed at a distance of 15 mm from an infected coral in an aquarium. Antonius [24] suggested that in the field, injured corals may become infected with BBD when located within a much larger distance of up to 1 m downstream from an infected coral. This suggestion corresponds with other studies [18], [27]–[28] who reported that corals infected with BBD are aggregated. In contrast, Edmunds [17] suggested that BBD-infected corals do not appear in aggregations, and therefore localized inter-colonial transmission is less likely. Another mechanism, recently suggested by Aeby and Santavy [29], is that the transmission of BBD may be vector mediated via corallivorous fish. However, their experimental data also show that tissue injury is a prerequisite for an infection to occur.

With few exceptions [e.g., 30], monitoring of coral diseases is carried out by generating snapshots of the disease prevalence and assessing the impact of the disease on coral populations or communities. Unfortunately, this monitoring scheme is usually not designed to provide quantitative epizootiological information. In a remarkable study, Jolles et al. [30] monitored the infection of the sea fan *Gorgonia ventalina* by the fungus *Aspergillus sydowii* across a relatively small spatial scale (200 m^2^). They analyzed the spatial distribution of this disease by using Ripley's *K* [31]–[32] as a measure of disease aggregation. Thereafter, they converted the spatial pattern of the disease into information about the transmission mechanism underlying the observed pattern. Their results suggest that both water-borne infection and secondary transmission by physical contact between the sea fans take place. Jolles et al. [30] demonstrated how the pattern of the spatial distribution of infected corals (as described by Ripley's *K* statistic), has the potential to reveal possible mechanisms of disease transmission in natural populations.

In contrast to *Aspergillosis*, which is a persistent disease with visible signs that do not change dramatically all year round, some other coral diseases are known to be strongly associated with high water temperature [e.g., 33]. Therefore, their prevalence and spatial pattern are seasonally dependent. In such a case, one snapshot is most probably insufficient for studying the dynamics of the disease and a repetitive monitoring scheme would be more suitable in order to infer processes, such as possible modes of disease transmission during the outbreaks. In this study, such a scheme was used to monitor the dynamics of BBD within a coral community. Similarly to Jolles et al. [30], we tested the spatial distribution of the disease across a relatively small spatial scale, where the underlying distribution of the susceptible corals was factored out from the analysis. However, since BBD is known to be associated with high water temperature and emerges during the warm months of the summer [17]–[18], [24], [26]–[27], [34]–[35], a repetitive monitoring scheme was used in order to enable characterization of the disease dynamics over both space and time.

**Figure 1:**
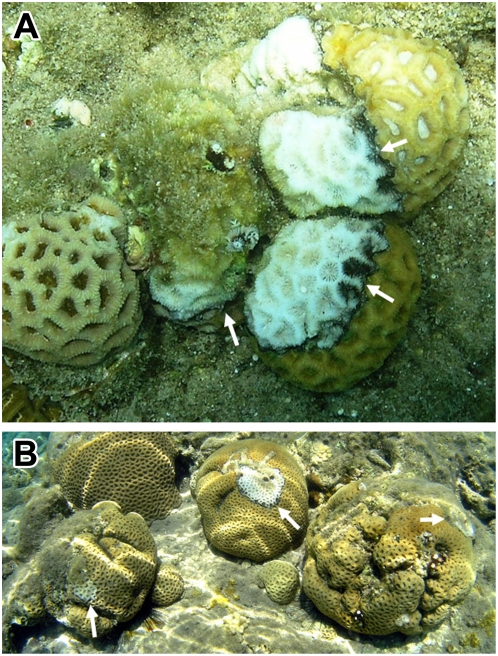
Example of clusters of three corals infected with black-band disease (BBD) from the genus *Favia*. (A) situated in direct contact, and (B) separated by a few centimeters from each other. The white arrows point to the black-band, defining the active location of the disease.

The different transmission mechanisms detailed above generate different predictions regarding the spatio-temporal distribution pattern of BBD within coral communities. In order to outline these predictions, the following terminology is introduced. We define newly-infected corals (NICs) as those showing signs of BBD that were not infected in the previous snapshot. Similarly, previously-infected corals (PICs) are corals that were infected in the previous snapshot. The direct contact mechanism, offered by Rützler et al. [26], predicts that NICs will be adjacent to the PICs, and together they will form a cluster of infected individuals that are in physical contact with one or more adjacent neighbors (e.g., Fig. 1A). The vector mediation mechanism, suggested by Aeby and Santavy [29], predicts different clustering patterns depending on the mobility of the vector. Vectors such as corallivorous fishes may form extensive clusters that may not be detected on a relatively small spatial scale. Alternatively, marine snails or fire worms (e.g., the fire worm *Hermodice carunculata*, a vector for the coral-bleaching pathogen *Vibrio shiloi*; [36]), which are more locally active, may hypothetically form clusters on a relatively small spatial scale. In addition, water-borne infection may also form clusters of infected corals, including corals that are not in direct contact with other corals (e.g., Fig. 1B). The microbial assemblage that forms the black-band in infected corals is loosely attached to the coral surface, and can easily be dislodged by the water movement adjacent to the coral [37]. In such cases, infectious material may be released into the water, drifting to nearby corals. This mechanism may form local infection clusters because the infection likelihood of a susceptible coral increases as the distance from the source/s of the infectious material (i.e., PICs) decreases.

In this study, we attempted to elucidate the transmission mechanisms of BBD within a coral community and to determine whether there is a detectable imprint of local transmission. To achieve this, we monitored the spatio-temporal distribution of susceptible corals as well as corals infected with BBD over a relatively small spatial scale in a natural coral community in Eilat (northern Gulf of Aqaba, Red-Sea).

## Methods

### Site selection

The reef chosen for observation is situated in shallow water (depth of ca. 1.5 m) off the shore of the Interuniversity Institute (IUI) in Eilat. Based on a flat beachrock, the reef is very uniform with respect to bathymetry and is situated on a gentle slope (ca. 3°). Although this area is shallow, exposed to wind action and therefore exposed to current, there is no dominant water-flow direction characterizing this reef since the local current is strongly affected by changing wave action. These bathymetric and oceanographic features help to avoid significant influences of reef microhabitat on disease clustering patterns. The coral community in this area is extremely dense (>50 corals/m^2^) and is composed of mostly massive corals, many of which are susceptible to infection by BBD. Since the density of susceptible corals is very high (>30 corals/m^2^; see ‘*Results*’), there is a relatively large number of infections per unit area (0.41 and 0.47 infected corals/m^2^ per year; see ‘*Results*’). This makes the area an ideal ‘natural laboratory’ for studying the spatial distribution and the dynamics of BBD within a natural community on a relatively small spatial scale.

### Field sampling

To study the dynamics of BBD in this community, a 10×10 m plot subdivided into one hundred 1 m^2^ squares was surveyed by snorkeling once a month, from July 2006 until December 2007. Using photography (photoquadrats), all susceptible corals within this area were mapped (similarly to Weinberg [38]) and an X-Y coordinate of the coral's centre within the quadrat was allocated (following the “*center rules*” scheme of Zvuloni et al. [39]). Once a month the location of infected corals was recorded, with corals being classified as infected if they showed the typical sign of BBD (i.e., a band-shaped black-to-red microbial mat; [40]). Continuous measurements of sea-surface temperature (SST), ca. 20 m away from the studied area, were received from the Israel National Monitoring Program of the Gulf of Eilat (NMP; http://www.iui-eilat.ac.il/NMP/).

### Spatial analysis of BBD

We made use of spatial statistics to characterize the spatial pattern of BBD-infected corals [41]. The Ripley's *K* index [31]–[32] was used to quantify non-random clustering patterns of infected corals within an area in terms of the degree and spatial scale of aggregation. Ripley's function 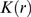 is defined as the expected number of infected corals within a radius *r* from an arbitrary infected coral. The function is normalized by dividing by the mean number of infected corals per unit area and is calculated as:
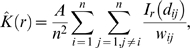
(1)where *A* is the total area of the site, *n* is the number of infected corals and *d_ij_* is the distance between any two infected corals *i* and *j*. The indicator variable 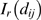 indicates whether or not there is an infected coral within radius *r* from coral *i*. Thus, 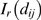 receives a value of 1 if *d_ij_*<*r* and 0 otherwise. Because the area under study is finite, portions of the circles having radius *r* might partially fall outside the site. To account for these border effects a weighting factor, 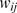, is introduced and defined as the proportion of the circumference of each circle which lies within the site [42].

A randomization test was devised to ascertain whether the *n* infected corals found in the field are significantly spatially aggregated, as compared to the aggregation found in the entire pool of the susceptible corals, by using a null hypothesis approach. The test made use of Besag's *L* function, 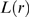 [43], which is Ripley's *K* index after appropriate transformation:
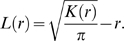
(2)Note that with this scaling, a variable that has a spatial distribution that is Poisson will result in the expected value of 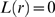 [43].

A null distribution for 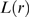 was generated as follows. A group of *n* corals was randomly chosen from the whole pool of susceptible corals without any discrimination as to whether individuals were healthy or infected. This was repeated 1,000 times so that 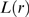 could be calculated for each group of *n* corals for any value of *r*. These results made it possible to calculate a 95% confidence interval (CI) envelope [*L_1_*(*r*), *L_2_*(*r*)] for *L*(*r*). We then calculated 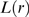 using only the infected corals found in the field and referred to this observed value as 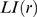. If 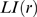 was found within the envelope [*L_1_*(*r*), *L_2_*(*r*)], then the distribution of infected corals was considered random, or at least not statistically different from the pool of the susceptible corals as a whole. Otherwise, if 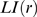 was found outside the envelope, the distribution of infected corals was considered significantly non-random compared to the entire pool of corals at *α* = 5% level. Infected corals were considered aggregated in distance scales where 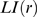 was found to be larger than *L_2_*(*r*), and over-dispersed where 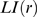 was found to be smaller than *L_1_*(*r*).

The above test was used to analyze the observations in each month for all corals that had shown active signs of BBD up to that point of time since the beginning of the disease season. Additionally, we used Ripley's *K* to test the spatial aggregation of all the corals that had died as a result of the disease and of those who survived (i.e., the coral was not recovered, but visible signs of BBD disappeared).

### Spatio-temporal analysis of BBD

To test whether local transmission (i.e., inter-colonial transmission within the studied site) is significant within the coral community in the course of disease outbreaks and establishments, we examined pairs of sequential sampling dates and tested whether NICs develop in proximity to PICs. Let 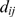 be the Euclidian distance between NIC *i* and PIC *j*. Then 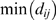 is the distance from NIC *i* to its nearest neighboring PIC from the previous month. The average distance between NICs in a given month to their closest PIC from the previous month, 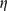, is given by:
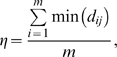
(3)where *m* is the number of NICs in a given month.

If the observed 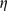 is found to be significantly smaller than would be expected had the disease infected random NICs independent of their spatial location, we can conclude that local transmission plays a role in the spread of the disease within the community. To test this hypothesis we generated 10,000 realizations of a particular snapshot. In each realization the group of PICs found in the field was held fixed, while *m* NICs were randomly selected from the pool of all susceptible corals, independent of their spatial location. 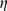 was then calculated according to Eq.3. The distribution of the 10,000 values of 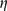 was considered to be a null distribution against which the observed 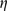 value could be tested. We used a two-tailed test with a significance level of 5%, to examine whether the observed 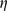 was significantly different.

In addition to testing the spatio-temporal dynamics of BBD from month to month within a season, we were interested in testing the dynamics between disease seasons. We examined whether surviving corals whose disease had stopped showing clinical signs of infection (i.e., observed expression of BBD) at the end of the first season of 2006, were more likely to become re-infected in the following season of 2007. If so, it would seem feasible that surviving corals from one disease season play a role in the re-introduction of the disease to the community in the following season. The test compares the observed number of re-infection events to those expected under a random infection scenario, where all susceptible corals in the area (including the survivors) have the same likelihood of being infected. A theoretical model based on the hypergeometrical distribution allows us to test for a significant difference (see Eq. 6 in the ‘*Results*’).

### A model of local water-borne disease transmission

We propose a model for the spatio-temporal transmission of the disease under the assumption that BBD is a water-borne infection. During an infection season, the model defines the likelihood that a susceptible coral in a given month will be infected by suspended infectious material originating from PICs within the study site. The likelihood is based on geometrical considerations and assumes that a susceptible coral has a probability of being infected by any PIC in a manner that is inversely proportional to the distance (*r*) of the PIC. Thus, the infection likelihood of any coral *i* is proportional to:
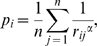
(4)where *n* is the number of PICs, 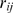 is the Euclidian distance between a given susceptible coral *i* to a PIC *j*, and *α* is an exponent that determines the decay of the transmission probability with distance.

The model predicts that NICs should be randomly located around the PIC, with a higher likeliness of being found nearby than further away. An assumption of the model (to be tested) is that this distance decay is distributed according to an inverse power-of-*α*-law scaling. NICs will tend to appear close to the PIC but have a small probability of being located far away (proportional to *1/r^α^*). Because there are often several PICs, the probability of susceptible coral-*i* being infected should be considered as the sum of the contributions from each PIC, namely 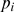.

We note that the methodology used to reconstruct the spatial probability distribution function is very similar to Kernel density estimation [44]–[45]. The assumptions underlying the construction of these models are that: (a) there is a preference for infections of nearby neighbors rather than distant ones; and (b) there is a cumulative impact of multiple infections on a single susceptible coral, such that the more infected neighbors a susceptible coral has, the more likely it is to become infected itself.

In order to test the 1*/r^α^* model of transmission we simulated the infection process at the studied site based on a given set of PICs for a particular date. Thus, infected corals from the first month in each pair of sequential sampling dates defined the *n* fixed PICs. Then, for a simulation that required generation of *m* NICs, we simply chose *m* corals at random from the entire pool of corals, assuming that coral-*i* has a probability of being chosen that is proportional to 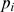. We repeated this process 10,000 times for *α* = 1, 1.5, 2 and 3 and compared the spatial distribution of NICs in these random realizations (the null distribution) to the spatial distribution of the observed NICs found in the field

To test the model the following statistic was employed:
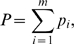
(5)where *m* is the number of NICs. We then calculated the *P* found in each realization and compared the distribution of the simulated *P'*s to the observed *P* found in the field. If the observed *P* was significantly different from the null distribution of the simulated *P'*s under a two-tailed test of 5% significance level, this would mean that the results found in the field do not agree with our proposed model. We carried out this test for each value of *α* and for all pairs of sequential sampling dates in which PICs and NICs appeared.

### Rate of spread

The rate of spread of BBD within the studied community was estimated by calculating the basic reproduction number 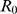 [46]. This index measures the epidemic potential of a pathogen and is defined as the mean number of secondary infections caused by a typical single infectious individual in a wholly susceptible coral community. When 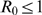, the introduction of an infected individual will fail to result in an outbreak, although it may lead to a localized infection. If, however, 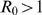, then it is possible for the introduction of the disease to result in an epidemic and the disease may persist for extended periods. For both of the studied disease cycles 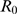 was calculated for the time period between June and August (the beginning of the infectious period in which inter-colonial infection was indicated; see ‘*Results*’). At the onset of an outbreak the cumulative incidence of infectives grows approximately exponentially with time (and therefore the incidence grows exponentially too; [47]). Following Roberts and Heesterbeek [47] we used the approximate relationship 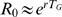 to estimate 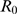. The parameter *r* is the exponential growth rate obtained by fitting an exponential function to the (cumulative) incidence, and *T_G_* is the observed mean generation interval of the epidemic.

## Results

### Impact of BBD in the study site

**Figure 2:**
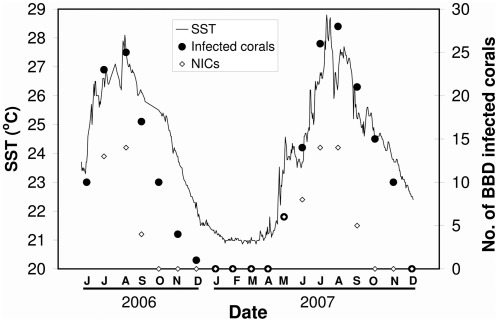
Number of corals infected with black-band disease (BBD) within the studied site, and sea-surface temperature (SST) starting from June 2006 to December 2007.

Within the 100 m^2^ plot, **3175** susceptible corals were observed and mapped. The surveyed community was composed of 73.8% corals belonging to the genus *Favia*, 13.1% *Platygyra*, 11% *Acanthastrea*, 1.9% *Favites* and 0.2% *Goniastrea*. The number of infected corals observed within the study site ranged from a low of zero during both winters to a peak of up to 25 infected corals in the summer of 2006 and another peak of 28 infected corals in the summer of 2007 (Fig. 2). The cumulative number of corals infected with BBD was 41 in 2006 and 47 in 2007 (1.3 and 1.5% of the susceptible corals, respectively). Among the 41 corals infected in the disease season of 2006, 24 (58.5%) corals died and 17 (41.5%) corals survived, after which the disease stopped showing any clinical signs. In the disease season of 2007 a similar pattern emerged, where 24 (51.1%) of the infected corals died and 23 (48.9%) survived. In total, 1.5% of the susceptible corals died over the two studied disease seasons.

### Seasonal pattern and rate of spread of BBD

BBD prevalence in the study site was found to be strongly associated with high water temperature (Fig. 2; *R^2^* = 0.86, *p*<0.001). The incidence of the disease (i.e., number of new individuals who contract a disease during a particular period of time) was also found to be associated with high water temperature (Fig. 2; *R^2^* = 0.58, *p*<0.001). During the survey the SST reached a seasonal maximum of 28.1°C in August 2006 and 28.8°C in August 2007, and a seasonal minimum of 20.8°C in March 2007. Our initial observations began in late June 2006, soon after the beginning of the disease season that year, where 10 corals had already been observed as infected. For the beginning of the infectious period, between June and August 2006, the introduction of the disease resulted in an epidemic-like growth with 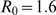 (*r* = 0.65; *T_G_* = 0.75). The highest level of disease prevalence (0.8%) and incidence rates (14 NICs per month) in the first studied season occurred in August 2006, two days after the SST reached a maximum. As of October 2006 the disease stopped spreading within the studied area and the number of NICs dropped to zero. By January 2007, when the SST dropped to 21.3°C, there were no corals with signs of the disease at the studied site, and by that point all the infected corals from the first studied season had either died or survived (showed no signs of the disease). Signs of BBD re-appeared only in early May 2007, when the SST reached 23.5°C. A similar seasonal pattern of the disease to that of 2006 was observed in 2007. For the time period between June and August 2007 the introduction of the disease also resulted in an epidemic-like growth with 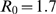 (*r* = 0.55; *T_G_* = 0.95). The highest level of disease prevalence (0.9%) and incidence rates (14 NICs per month) in the second studied season occurred in August 2007, 19 days after the SST reached its maximum point. Similar to the previous season, by October 2007 NICs were not observed in the studied site and by December 2007 all the infected corals had either died or survived.

### Spatial pattern of BBD

Ripley's *K* test was first applied to check the distribution of the entire pool of susceptible corals in the studied site. This tests the null hypothesis that the corals are randomly distributed, having a Poisson distribution [i.e., *L*(*r*) = 0]. As *L*(*r*) was found to be significantly greater than zero (*p*<0.01) for all *r'*s, the pool of the susceptible corals should be considered aggregated over the entire tested range of spatial scales.

**Figure 3:**
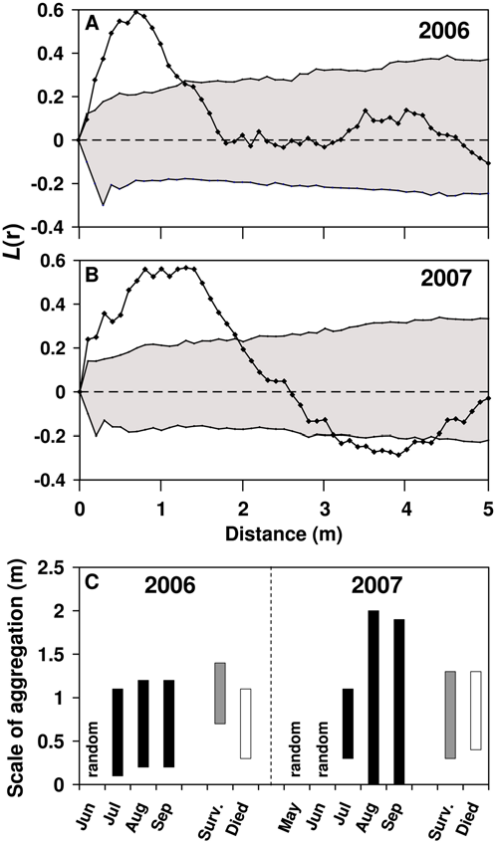
Spatial patterning of black-band disease (BBD). (A) and (B) Besag's *L* plots for all corals infected by BBD throughout the disease seasons of 2006 and 2007, respectively. The dashed line (*L* = 0) represents a spatially random (Poisson) distribution. Line with dots represents observed *L* values (Eq. 2) for infected corals. Shaded area represents the Monte Carlo 95% confidence interval (CI) envelope (see text). For distance scales (*r*) where *L* values fall within the envelope, the spatial distribution of infected corals does not differ significantly from the distribution of the susceptible corals as a whole. Infected corals are significantly aggregated (/over-dispersed) where the observed *L* values fall above (/below) the CI envelope. (C) Distance scales where the observed *L* values are above the range given by the 95% CI envelope. Black bars represent scale of aggregation of the accumulated corals infected by BBD for any month during the disease spread. Grey bars represent scale of aggregation for all surviving corals whose disease state was no longer evident (i.e., visible signs of BBD disappeared) at the end of the disease season. Finally, white bars represent scale of aggregation for all corals that died due to the disease by the end of the disease seasons.

We then tested the spatial distribution of BBD-infected corals. By the end of the first season (January 2007), when signs of BBD were no longer observed, the accumulated corals which had been infected by BBD during the first season (including those that had died and those that had been infected and survived) were examined and found to be aggregated on small spatial scales of 0.2–1.2 m compared to the null distribution of the susceptible corals (Fig. 3A). Under this distance, affected corals could be grouped into nine clusters containing several corals, in addition to five single corals. During the first studied season there were six potential incidents of transmission between corals that were in direct contact (e.g., Fig. 1A) and 21 incidents of transmissions between corals, which formed clusters of infected corals that were not in direct contact (e.g., Fig. 1B). A similar pattern was found at the end of the second season (December 2007). Here, the accumulated corals which had been infected by BBD in the season of 2007 were aggregated at small spatial scales of up to 1.9 m (Fig. 3B). There were eight incidents of transmission by direct contact and 30 potential incidents of non-direct contact transmissions. Under this distance, affected corals could be grouped into six clusters containing several corals and two single corals.

Fig. 3C represents results of 13 Ripley's *K* analyses as carried out for both years. It shows distance scales where the observed *L* values (Eq. 2) are above the range given by the 95% CI envelope [i.e., 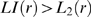]. Whenever this occurs, the coral distribution may be considered non-random, showing strong features of aggregation compared to the natural aggregation of the susceptible corals. Black bars represent scale of aggregation for the accumulated infected corals for each of the nine months of disease spread (4 in 2006 and 5 in 2007). In June 2006 and in May and June 2007, the spatial distribution of BBD among the corals did not differ significantly from the spatial pattern of the susceptible corals as a whole. However, from July to September in both years, when the prevalence of the disease increased (Fig. 2), the infected corals were found to be significantly more aggregated than the null distribution. Additionally, in both years the accumulated corals that died due to the disease (white bars) and the surviving corals (grey bars) also showed spatial aggregation over a relatively small spatial scale of up to 1.3 and 1.4 m, respectively. In the supplementary materials we provide, as an example, a spatial illustration of the disease dynamics over the 2007 disease season (Illustration S1).

### Spatio-temporal pattern of BBD

**Figure 4:**
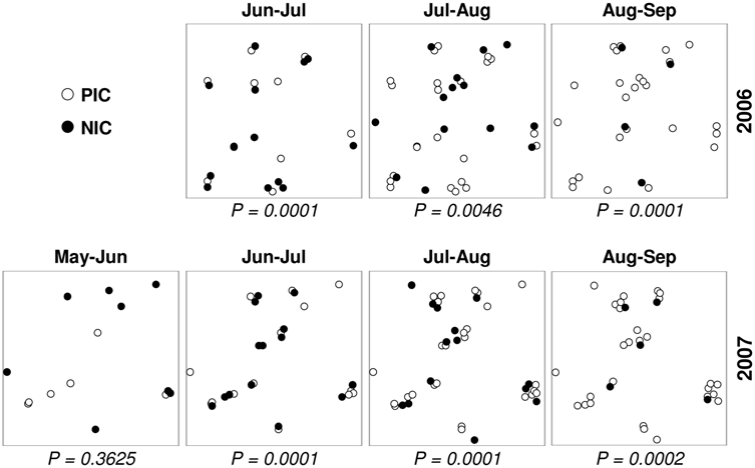
Spatial distribution of newly-infected corals (NICs; full circles) in relation to previously-infected corals (PICs; empty circles) within the studied site. The *p*-value for each pair of sequential sampling dates is associated with the mean minimum nearest neighbor statistic, 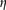 (Eq. 3). The null hypothesis is that the average 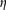 for a given pair is not significantly different from that expected had the infection transmitted randomly within the susceptible corals with equal probability for all corals. *p*-values smaller than 0.025 show that there is a significant deviation from random infection and, in such cases, NICs are found to develop in significant proximity to PICs.

We tested the spatio-temporal distribution of NICs in relation to PICs of the previous month using the *η* statistic defined in Eq. 3. Our null hypothesis was that the mean distance between NICs in a given month to their nearest neighboring PIC from the previous month would not be significantly different than that expected if all susceptible corals had an equal probability of being infected. The results showed that during the beginning of the disease seasons of both years, NICs appeared to be significantly closer to PICs than would be expected by chance (Fig. 4). That is, in nearly all cases the hypothesis that the NICs were infected by a random process of disease transmission independent of the spatial location of the PICs was rejected. There was one exception to this in June 2007.

It is interesting to note that there was a significantly high probability that previously surviving corals would be re-infected at the beginning of the following season. Seventeen of the 3,151 susceptible corals at the beginning of the disease season of 2007 were corals that had survived BBD infection during the previous season. Five of the 17 surviving corals (29.4%) were re-infected, within a pool of 47 infection cases in 2007 (three in May and two in June). According to combinatorial considerations, the probability that *R* re-infections might occur by chance [Pr(*R = k*)] is given by:
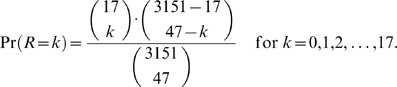
(6)The probability that the five or more re-infections occured by chance is calculated as:
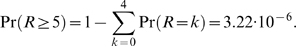
(7)Since 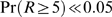, we conclude that the observed number of five re-infections is significantly unusual.

### A model of local water-borne disease transmission

**Figure 5:**
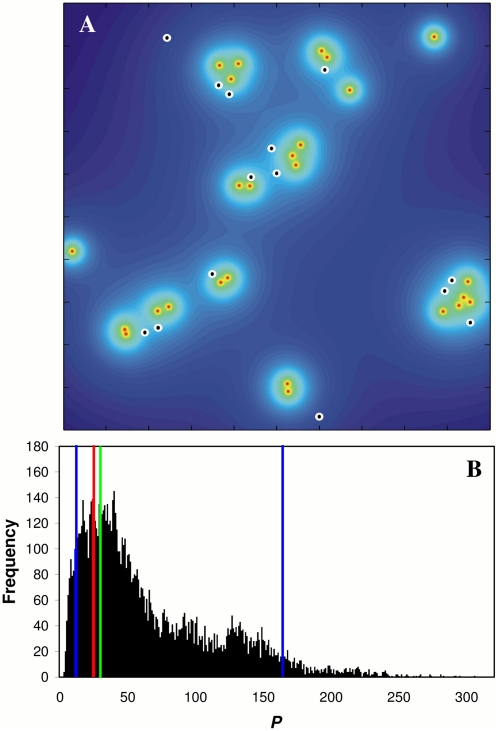
A 1*/r^2^* model of water-borne disease transmission. (A) Probability surface plot. An example of the probability of infection for each point at the studied site as calculated by Eq. 4 from a set of *n* = 26 infected corals observed in the field in July 2007 (red points). The probability of infection is displayed as a gradient of colors, such that warm colors (e.g., red) represent a high probability of infection and cold colors (e.g., blue) represent a lower probability of infection. The white circles with the black centers represent the newly-infected corals (NICs) observed one month later in August 2007, which are found to lie in close proximity to the ‘hot spots’ of infection (infected corals from the previous month). (B) An example of a histogram of 10,000 simulated *P'*s as calculated by Eq. 5 for *n* = 26 infected corals observed in July 2007 and *m* = 14 infected corals selected at random from the entire pool of corals. The simulations assume that coral-*i* has a probability of being chosen that is proportional to 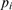 as calculated by Eq. 4 (for *α* = 2). The average simulated *P* was 30.2 (marked by vertical green line) and the *P* calculated for the *m* = 14 NICs observed in August 2007 was 23.9 (marked with vertical red line), within the range of the 95% confidence interval envelope (10.4 to 164; marked with vertical blue line) predicted by the simulations (*p* = 0.21).

Recall that the model simulates the spatial distribution of NICs, assuming that infections spread to susceptible corals at distance *r* from PICs with probability proportional to 1/*r^α^* (as defined by Eq. 4). After studying the distribution of *P* (Eq. 5) for repeated model realizations, the hypothesis that the observed NICs were produced by such a process could not be rejected on all occasions (i.e., for all pairs of sequential sampling dates) for *α* = 1.5 (*p*>0.32) except for May–June 2007 (*p* = 0.026). The same was true for *α* = 2 (*p*>0.21; except for May–June 2007 with *p* = 0.019). In contrast, the hypothesis was nearly always rejected for *α* = 1 and *α* = 3 (average *p*<0.042 and 0.036, respectively). As an example, Fig. 5A shows the probability of infection for each point in the studied site as calculated by Eq. 4, where *α* = 2, from a set of *n* = 26 infected corals observed in July 2007. Based on the same data set, Fig 5B shows a histogram for *P* (Eq. 5) obtained from 10,000 model realizations. In each realization *m* = 14 NICs were selected at random from the entire pool of corals with a probability that is proportional to 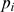 (Eq. 4) for *α* = 2. The average simulated *P* was 30.2 (vertical green line) and the observed value of *P* calculated for the 14 NICs found in the field in August 2007 was *P*_(obs)_ = 23.9 (vertical red line; *p* = 0.21). *P*_(obs)_ lies within the 95% CI (10.4 to 164; vertical blue lines) as generated by the simulations and, therefore, we could not reject the null hypothesis of water-borne infection.

## Discussion

Similar to reports from other locations [17]–[18], [24], [26]–[27], [34]–[35], BBD in Eilat is strongly associated with high water temperature (Fig. 2). The disease prevalence, which is relatively low even at its peak, and the spatial pattern of the disease are seasonally dependent. Consequently, in order to study the dynamics of BBD effectively, its spatial pattern within a studied site should be monitored repetitively throughout the year.

For both of the studied disease cycles the outbreak of BBD generated sustained growth of infected corals similar to an epidemic. The reproductive number 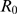 was calculated for the time period between June and August (the beginning of the infectious period) and was found to be greater than unity (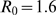 and 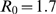 for 2006 and 2007, respectively). Although the two observed outbreaks generated epidemic-like growths, the prevalence of the disease remained low even at the peak of the disease season. We assume that the seasonality of the disease and its transmission, which may be driven by parameters that are changed in accordance to the season (e.g., water temperature and solar radiation), prevent the disease from spreading over a larger number of susceptible corals. However, in light of the predicted warming of the ocean, the disease season may, in the not too distant future, become longer with epidemics having higher prevalence then those observed here.

### Inference of spatial process from spatio-temporal pattern

At the beginning of the outbreak in both studied seasons, when the number of infected corals was relatively low, the spatial distribution of the disease among the corals did not differ significantly from the spatial pattern of the susceptible corals as a whole. However, clusters of infected corals began to emerge from July and continued until September (Fig. 3C). The number of infected corals in each cluster increased toward the peak of the disease season. In addition, testing the spatio-temporal relation between NICs and PICs showed that from July to September the appearance of NICs was strongly dependent on the spatial location of the PICs (Fig. 4). This clearly suggests that, during an outbreak, transmission of the disease is likely to occur from PICs to NICs. In other words, PICs are likely to be the source of infection for the NICs. Our suggested model of disease transmission strengthens this hypothesis. By defining an infection probability surface as a function of PICs (e.g. Fig. 5A), we find that the appearance of the disease is distributed around the PICs in accordance with a 1*/r^α^* law, where nearest neighbors tend to be infected first. The distribution of the NICs found in the field does not deviate from the expected distribution generated by this probability surface, thus demonstrating the high dependence of the appearance of NICs on PICs. As the model was not rejected for *α* = 1.5 and *α* = 2 (e.g., Fig. 5B), but rejected for *α* = 3, we conclude that the distance decay of the disease transmission is not as steep as 1/*r* ^3^. However, as the model was rejected also for *α* = 1, we believe that the distance decay is sharper than 1*/r*, indicating that the most important disease transmission route is via nearby neighbors.

In general, there are a number of possible transmission mechanisms that govern the spread of the disease. These include transmission by direct contact, water-borne transmission and vector-mediated transmission. We identified a total of 14 cases of transmission by direct contact over the course of two disease seasons. However, distinguishing between water-borne transmission and vector mediated transmission is generally not straightforward. Over the two seasons we observed a total of 43 infections in which NICs appeared in close proximity to PICs, but were not in direct contact with each other (i.e., the NIC and the PIC were not physically touching one another, see fig 1B as an example case). These incidents may be the outcome of either water-borne infection or of a vector-mediation mechanism. Since potential vectors with limited mobility (i.e., snails, fireworms) were not identified at the studied site (both at day and night), it is reasonable to assume that water-borne infection is a significant transmission mechanism of BBD over a relatively small spatial scale of up to 1.9 m. Our suggested model of water-borne disease transmission strengthens this hypothesis.

Our results contrast with those of Edmunds [17], who failed to find aggregations of BBD in the Virgin Islands, but are consistent with other studies [e.g., 18], [27]–[28] that proposed that corals infected with BBD appear to be aggregated. However, it is important to note that in the latter studies the underlying distribution of the susceptible corals was not factored out from the spatial analyses (an essential feature that has to be taken into account when analyzing the spatial pattern of diseases). Therefore, the reported aggregations of corals infected with BBD might also be an outcome of the natural spatial distribution of the susceptible corals at the studied sites. Our suggested transmission mechanism of the disease is in line with Richardson [37] and Bruckner et al. [27], who proposed that developed BBD bacterial mat can be easily dislodged into the water column by water movement. We assume that corallivorous fishes, which were suggested by Aeby and Santavy [29] to be a vector of BBD, did not contribute to the observed aggregations of BBD within our studied site. Such vectors that forage over a larger scale than the size of our studied site may form very extensive clusters that may not be detected on a relatively small spatial scale.

Although we observed that outbreaks had begun by June 2006 and in May 2007 in each of the disease seasons, infected corals did not form significant aggregations over the tested spatial scale until July in both years (Fig. 3C). It was also unusual that the NICs in June 2007 were not found to be aggregated in proximity to the PICs of May 2007, as distinct from all other pairs of sampling dates. These results could be an outcome of the low number of NICs, which characterizes the beginning of disease seasons, making it difficult to obtain statistical significance. Another possibility is that the initial invasion of BBD at the beginning of seasons originates from external sources of infection entering the area and randomly infecting susceptible corals, while aggregations build up later as a result of local infections (PICs to NICs).

However, similar to the findings of Rodríguez and Cróquer [48], in the disease season of 2007 the number of re-infected corals was significantly higher than expected by random. This indicates that corals which had previously been infected are more susceptible to being re-infected, in contrast to susceptible corals with a healthy history. Another possibility is that BBD surviving corals failed to heal completely and might have acted as ‘winter reservoirs’ of the disease, allowing it to persist throughout the winter but in an inactive state. In such a case, once the SST begins to rise towards May and June the infection re-emerges, showing the characteristic signs of BBD. It is important to mention that all cases of re-infection occurred only in the early stages of the disease season (three in May and two in June). This makes the possibility of random invasion at the beginning of seasons less likely and strengthens the hypothesis that these corals had never healed completely and thus act as ‘winter reservoirs’. In addition, the fact that these corals had indeed survived the disease season of 2006 implies that it is unlikely that their re-infection is due to their belonging to a genotype relatively more susceptible to BBD.

Transmission from PICs to NICs was not found to occur in May–June 2007 and the proposed model of local water-borne disease transmission was rejected for all *α'*s at this time. We suggest that mechanisms other than local water-borne transmission may act during the initial invasion of the disease. These mechanisms may include, for example, revival of BBD in surviving corals from the previous year.

### Annual cycle of BBD

We suggest that the dynamic of BBD in the Eilat coral community follows four main phases over the course of a year: (a) *Introduction* - from May to June, when SST starts rising, the infection enters either from external sources or possibly from internal sources (i.e., ‘winter reservoirs’); (b) *Establishment* - from July to September the prevalence of the disease increases and is associated with the SST reaching its peak. In this phase water-borne and direct contact are the most likely transmission mechanisms, leading to spatially aggregated infections; (c) *Regression* - from October to December/January, when SST starts dropping, NICs are not further observed and the PICs either die or survive; (d) *Silence* – from January to April, when SST is low, signs of the disease are no longer recognized but some of the surviving corals might be acting as ‘winter reservoirs’ of the disease within the community.

The manner in which seasonality affects the transmission pattern of BBD and the rate of new infections is not fully understood and requires future study, and is undoubtedly complex as in many other seasonally forced diseases [49]. However, key factors such as water temperature, radiation and nutrient concentration all change with the season. These factors may affect the behavioral responses of the BBD pathogens and as such govern the transmission pattern of the disease. It is possible that during the *Introduction* phase the black-banded bacterial mat is yet to be well developed and therefore it cannot easily be dislodged into the water column. Only later on, during the *Establishment* phase, when the black-banded bacterial mat becomes thicker it sloughed off into the water more easily and is transmitted to nearby corals by the water movement.

### Alternative explanations for the observed pattern

An alternative non-transmission mechanism explanation for the observed locally clustered patterns of BBD could be associated with variability of disease resistance between different species or different genotypes within each species. If the levels of susceptibility differ between the species we observed or between genotypes, and if species or genotypes with high susceptibility tend to grow relatively close to each other, this could also contribute to disease aggregations. However, the species we have been following are all massive corals, known to be spawners, and do not normally recruit in proximity to each other, nor do they form clonal propagules on such small spatial scales. Moreover, we found that eight of the 15 aggregated clusters of infected corals in both years included more than one species and even more than one genus within each cluster. This result makes this alternative explanation even less likely, since most of the clusters are not homogenous.

The reef chosen for this study was selected, among other things, because of its uniform and flat bathymetry. These features provided us with a model reef devoid of significant influences of microhabitat on disease clustering patterns. However, since this reef is unique in its particularly dense community of susceptible corals, it is possible that the observed dynamics of BBD may not be the same in reefs where coral communities are less dense and the bathymetry is more complex. An influence of coral host density on disease transmission was proposed by Bruno et al. [50] for outbreaks of white syndrome in the Great Barrier Reef. They suggested that high density reduces the distance between neighboring corals and thus between infected and healthy corals. As such, it increases the potential for disease transmission between corals in close proximity.

To summarize, similar to Jolles et al. [30], we found that spatial statistics combined with null hypothesis approaches are very effective tools for understanding epizootiological processes in coral reefs. In particular, Ripley's *K* function is specifically tuned to detect aggregations and this is the hallmark signature for the presence of localized transmission dynamics we seek to identify among the infected corals. Additionally, since the prevalence and spatial pattern of BBD are seasonally dependent, a combined spatio-temporal analysis was essential in order to properly assess the transmission pattern of the disease. Using such spatio-temporal analyses we show that: (a) local transmission, often not by direct contact alone, is an important factor in the spread of BBD within the studied community; (b) corals that were once infected but nevertheless survived appear to play a role in the re-introduction of the disease to coral communities after the *Silence* phase; and (c) water-borne infection is likely to be a significant transmission mechanism of BBD. Accordingly, there is a preference for infection of nearby neighbors rather than more distant ones, where the more infected neighbors a susceptible coral has, the more likely it is to become infected itself. The above findings, which are based on data collected in the field, answer some fundamental questions regarding epizootiological processes of BBD, the most widespread and destructive of coral infectious diseases. Further research should focus on how biotic and abiotic environmental factors influence the transmission pattern of the disease.

## Supporting Information

Illustration S1 Illustration of the dynamics of BBD over the studied site (10×10 m), during the disease season of 2007.
(0.19 MB PPT)
