# Supplementary material for: Spatio-Temporal Transmission Patterns of Black-Band Disease in a Coral Community
Source: PLoS One. 2009 Apr 1;4(4):e4993. doi: 10.1371/journal.pone.0004993 (PMC2660573; doi:10.1371/journal.pone.0004993)
Supplement: Illustration S1 — Illustration of the dynamics of BBD over the studied site (10×10 m), during the disease season of 2007. (0.19 MB PPT) [file pone.0004993.s001.ppt]

## Slide 1
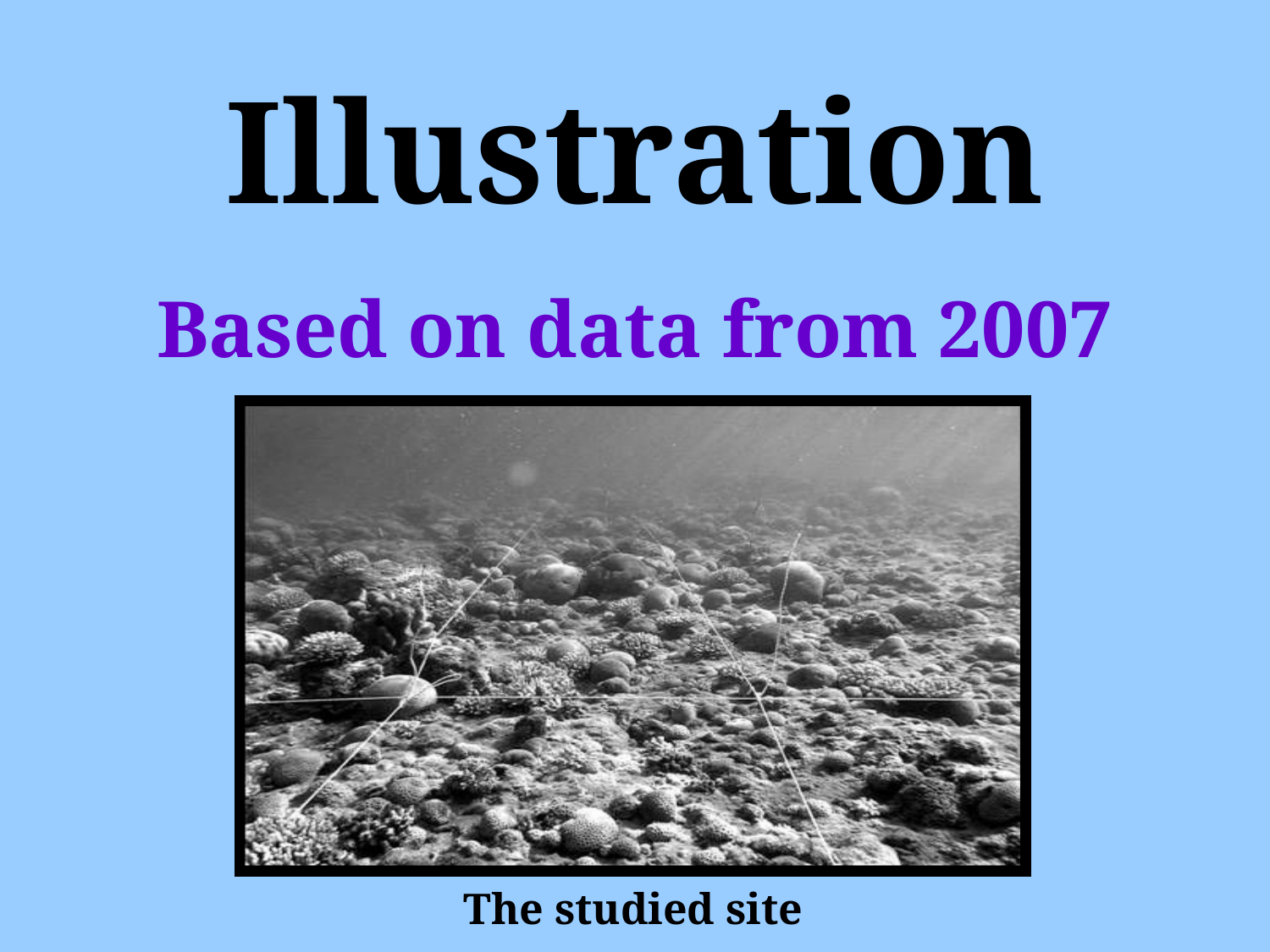

Illustration
Based on data from 2007
The studied site

## Slide 2
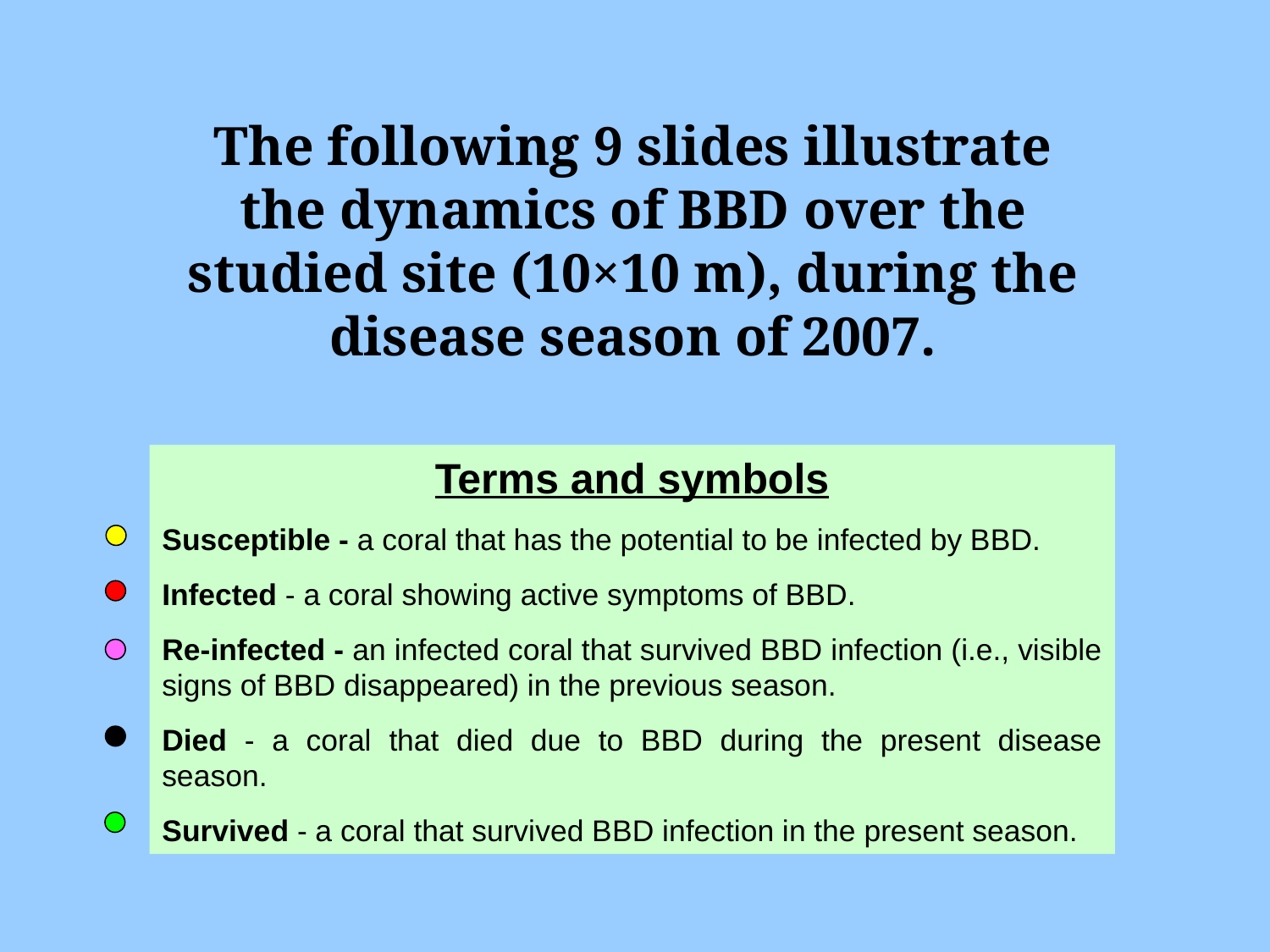

The following 9 slides illustrate the dynamics of BBD over the studied site (10×10 m), during the disease season of 2007.
Terms and symbols
Susceptible - a coral that has the potential to be infected by BBD.
Infected - a coral showing active symptoms of BBD.
Re-infected - an infected coral that survived BBD infection (i.e., visible signs of BBD disappeared) in the previous season.
Died - a coral that died due to BBD during the present disease season.
Survived - a coral that survived BBD infection in the present season.

## Slide 3
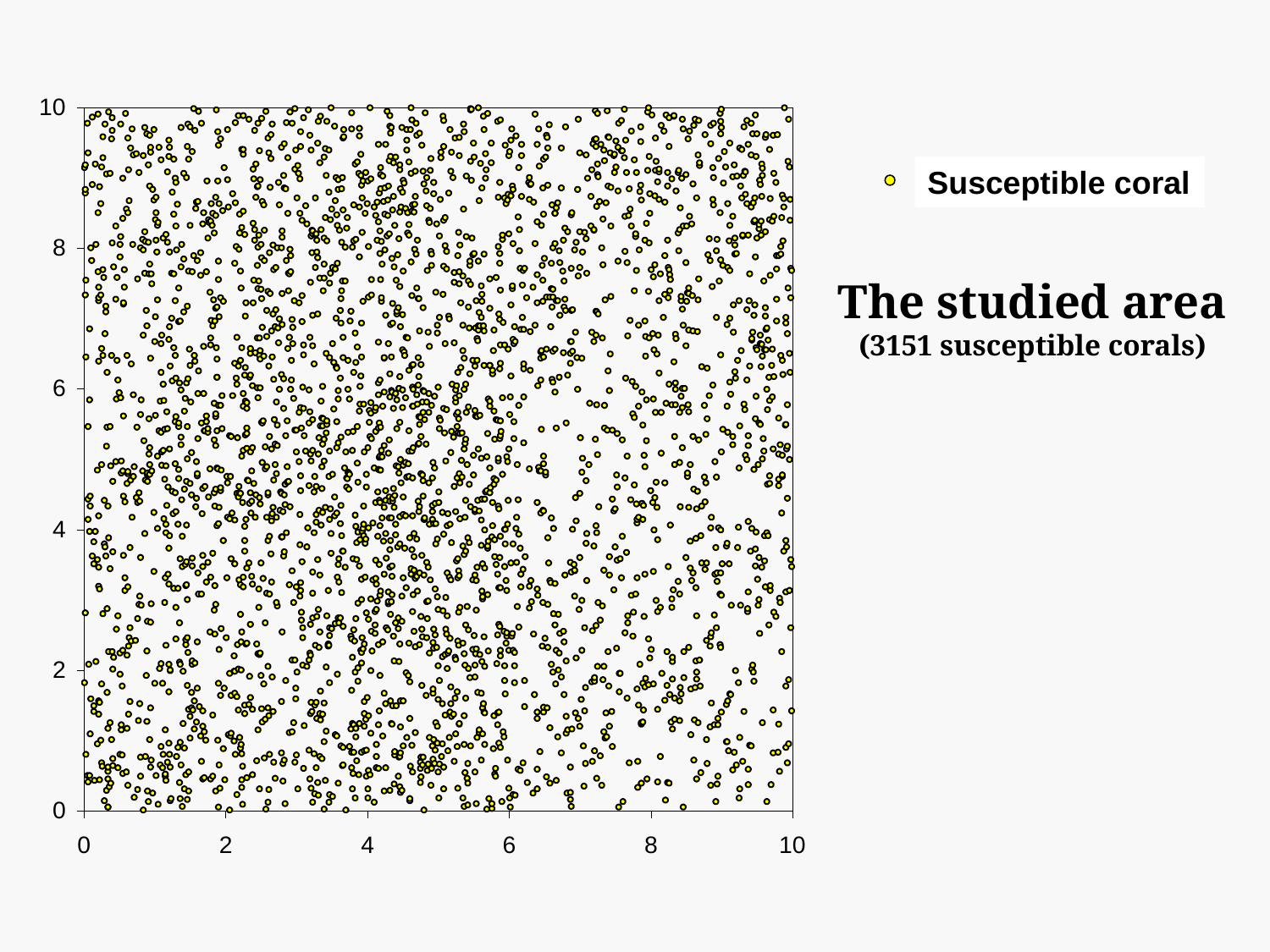

Susceptible coral
The studied area (3151 susceptible corals)

## Slide 4
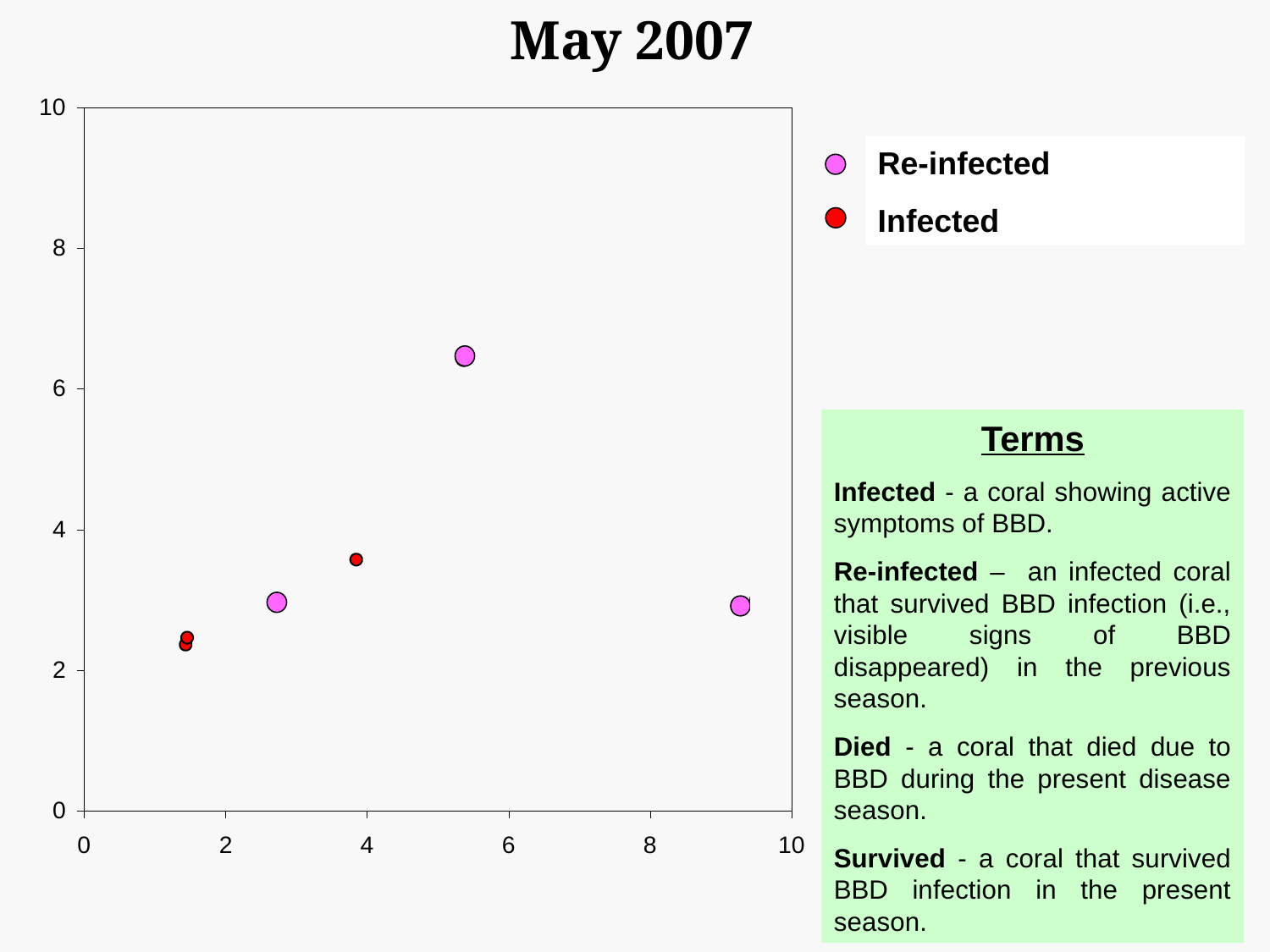

May 2007
Re-infected
Infected
Terms
Infected - a coral showing active symptoms of BBD.
Re-infected – an infected coral that survived BBD infection (i.e., visible signs of BBD disappeared) in the previous season.
Died - a coral that died due to BBD during the present disease season.
Survived - a coral that survived BBD infection in the present season.

## Slide 5
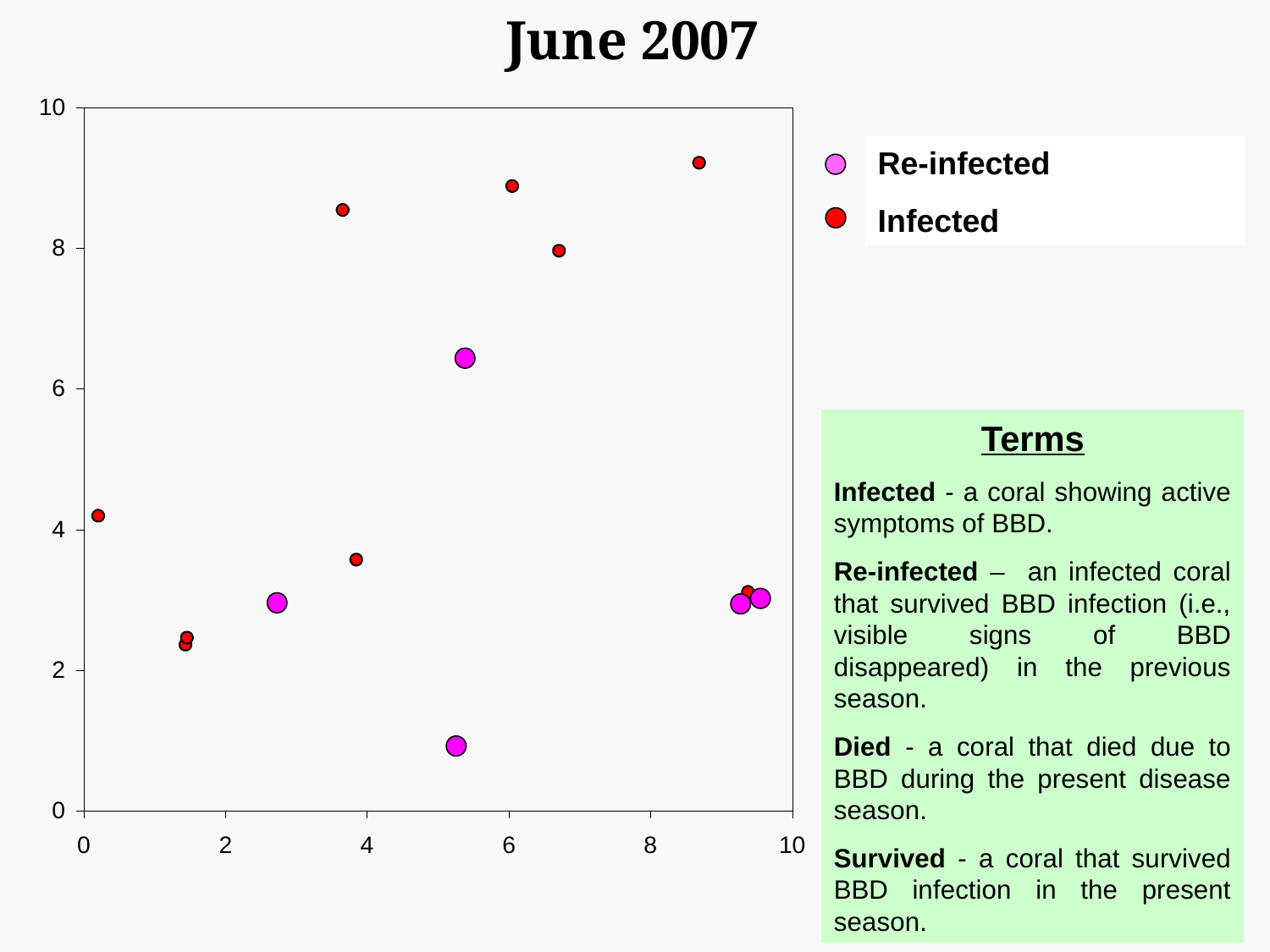

June 2007
Re-infected
Infected
Terms
Infected - a coral showing active symptoms of BBD.
Re-infected – an infected coral that survived BBD infection (i.e., visible signs of BBD disappeared) in the previous season.
Died - a coral that died due to BBD during the present disease season.
Survived - a coral that survived BBD infection in the present season.

## Slide 6
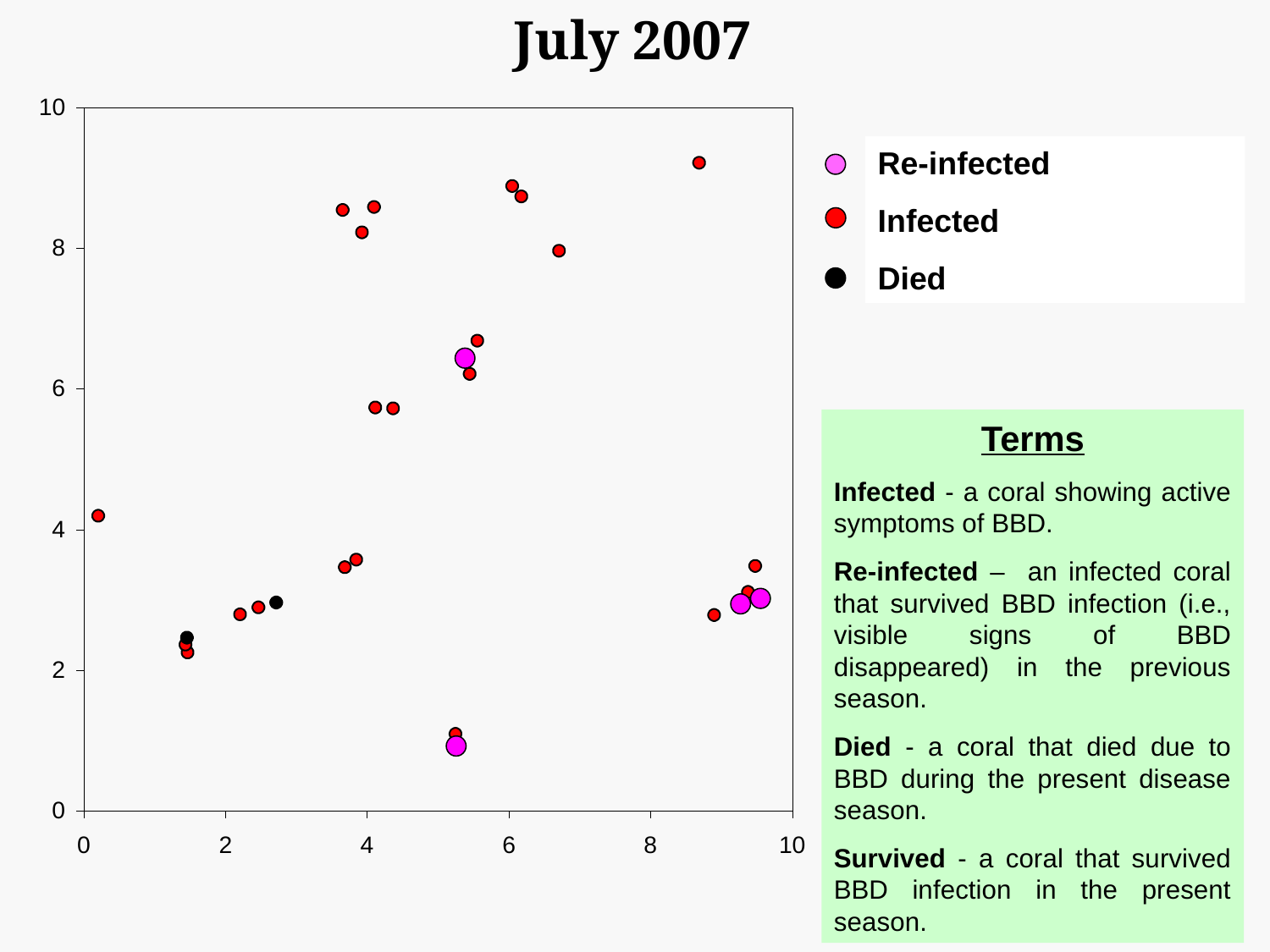

July 2007
Re-infected
Infected
Died
Terms
Infected - a coral showing active symptoms of BBD.
Re-infected – an infected coral that survived BBD infection (i.e., visible signs of BBD disappeared) in the previous season.
Died - a coral that died due to BBD during the present disease season.
Survived - a coral that survived BBD infection in the present season.

## Slide 7
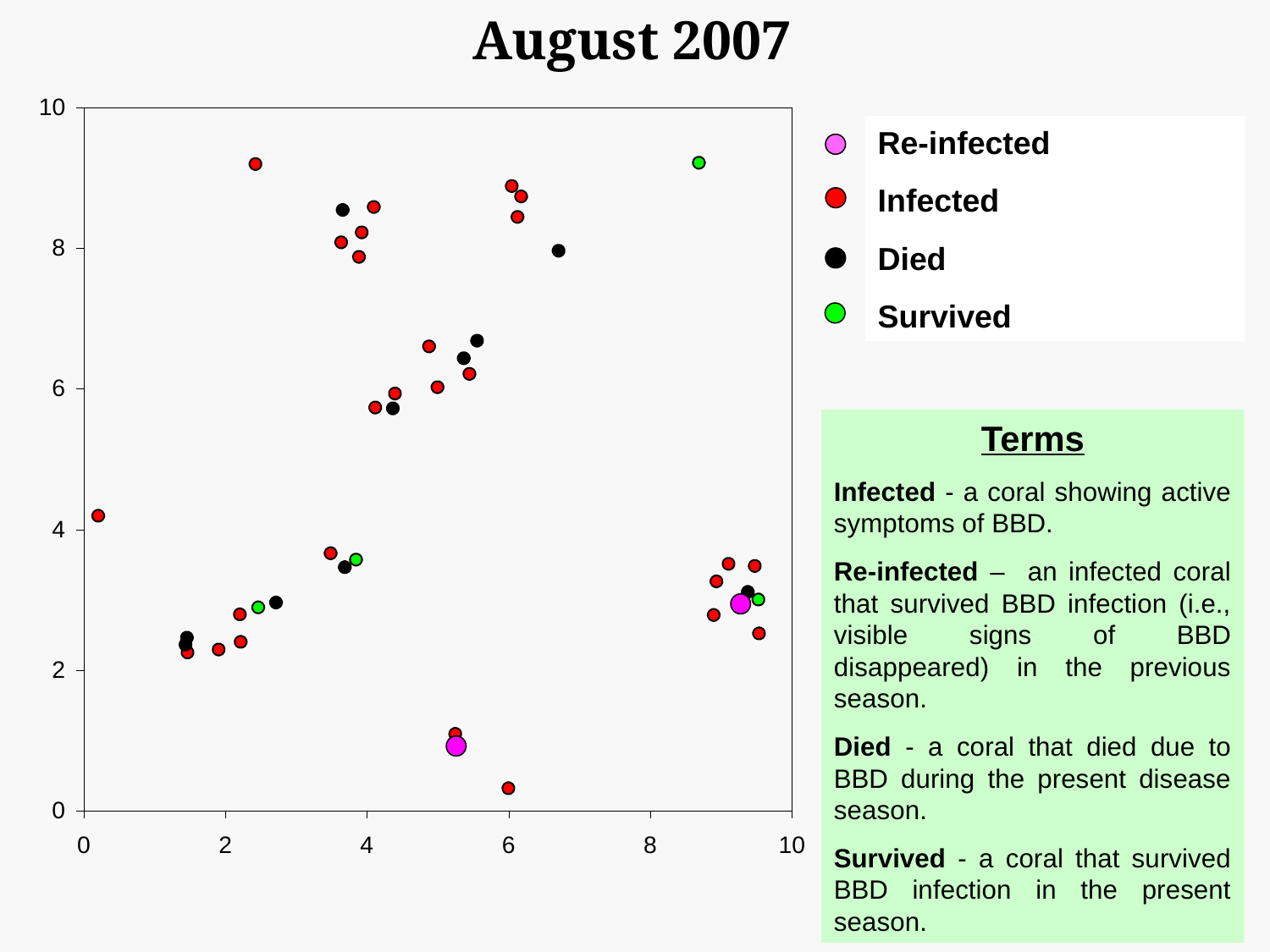

August 2007
Re-infected
Infected
Died
Survived
Terms
Infected - a coral showing active symptoms of BBD.
Re-infected – an infected coral that survived BBD infection (i.e., visible signs of BBD disappeared) in the previous season.
Died - a coral that died due to BBD during the present disease season.
Survived - a coral that survived BBD infection in the present season.

## Slide 8
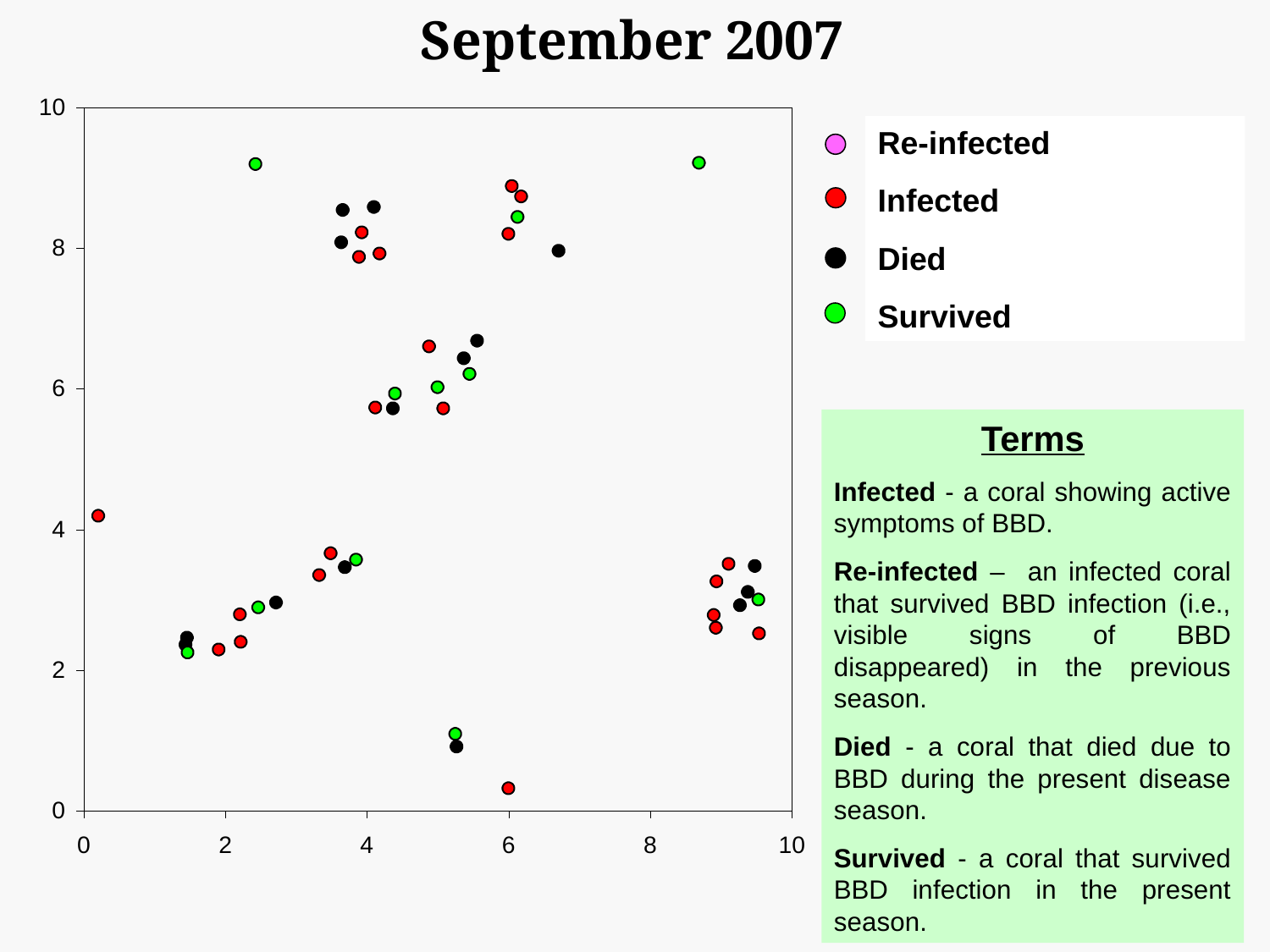

September 2007
Re-infected
Infected
Died
Survived
Terms
Infected - a coral showing active symptoms of BBD.
Re-infected – an infected coral that survived BBD infection (i.e., visible signs of BBD disappeared) in the previous season.
Died - a coral that died due to BBD during the present disease season.
Survived - a coral that survived BBD infection in the present season.

## Slide 9
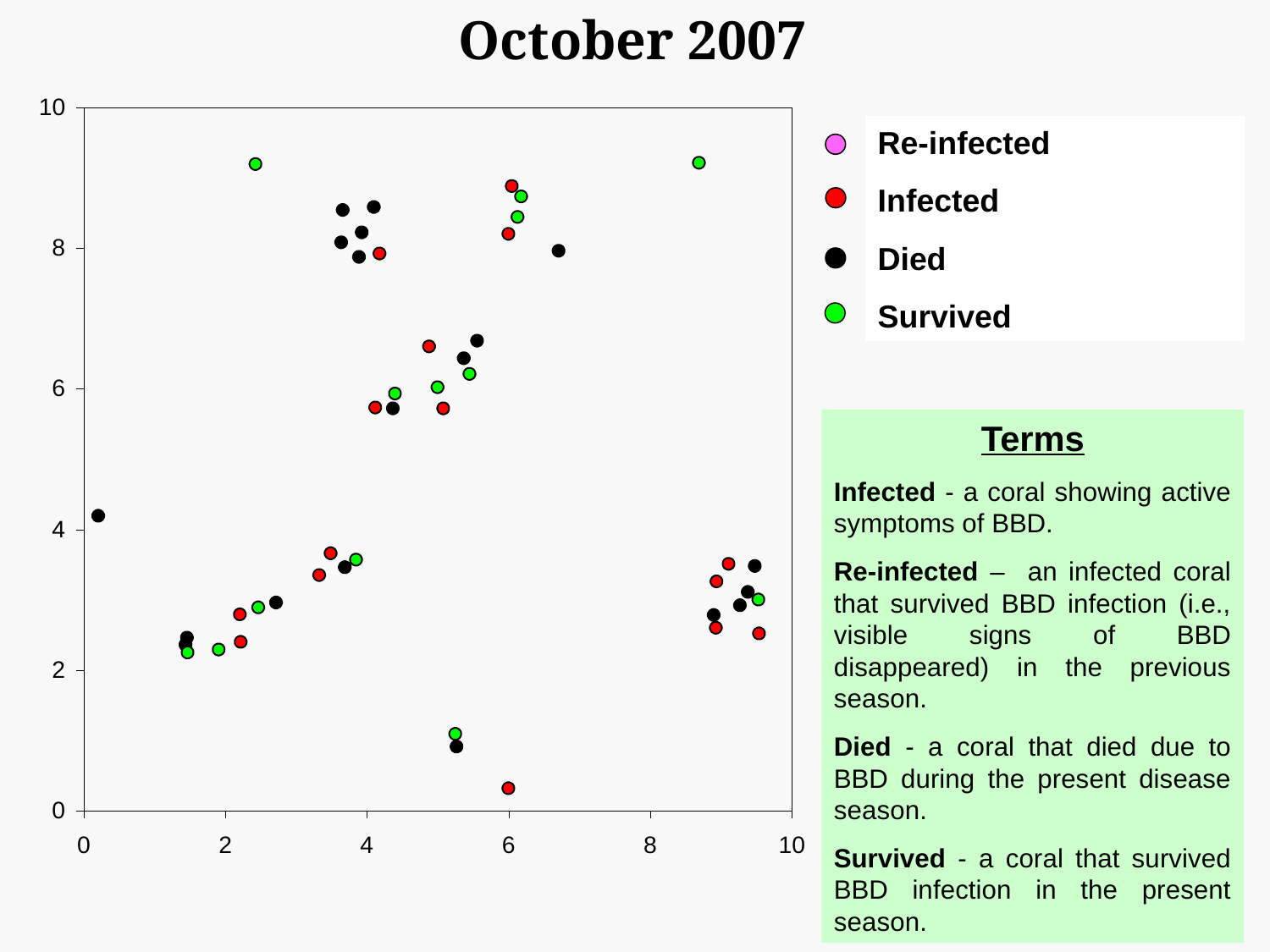

October 2007
Re-infected
Infected
Died
Survived
Terms
Infected - a coral showing active symptoms of BBD.
Re-infected – an infected coral that survived BBD infection (i.e., visible signs of BBD disappeared) in the previous season.
Died - a coral that died due to BBD during the present disease season.
Survived - a coral that survived BBD infection in the present season.

## Slide 10
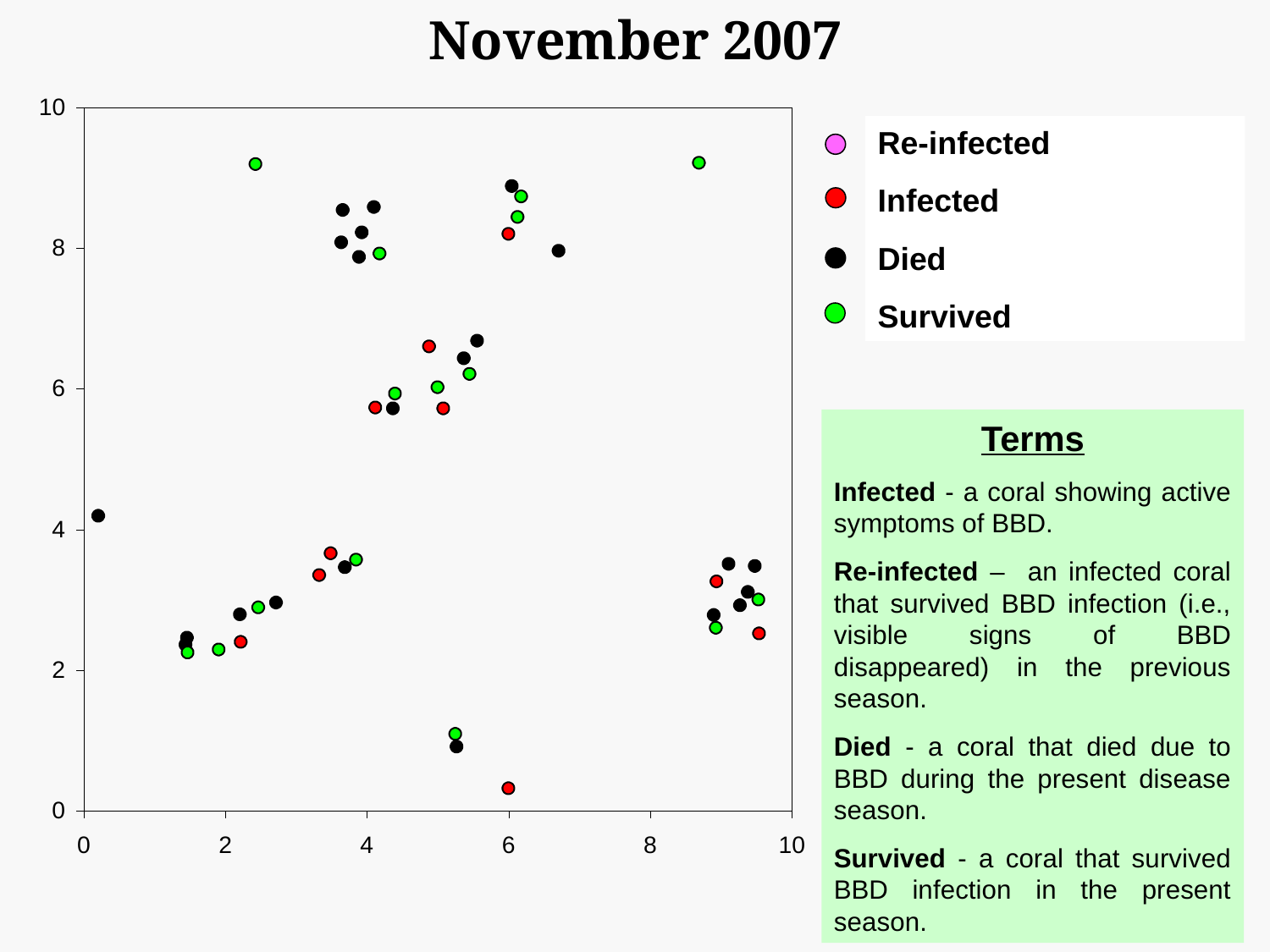

November 2007
Re-infected
Infected
Died
Survived
Terms
Infected - a coral showing active symptoms of BBD.
Re-infected – an infected coral that survived BBD infection (i.e., visible signs of BBD disappeared) in the previous season.
Died - a coral that died due to BBD during the present disease season.
Survived - a coral that survived BBD infection in the present season.

## Slide 11
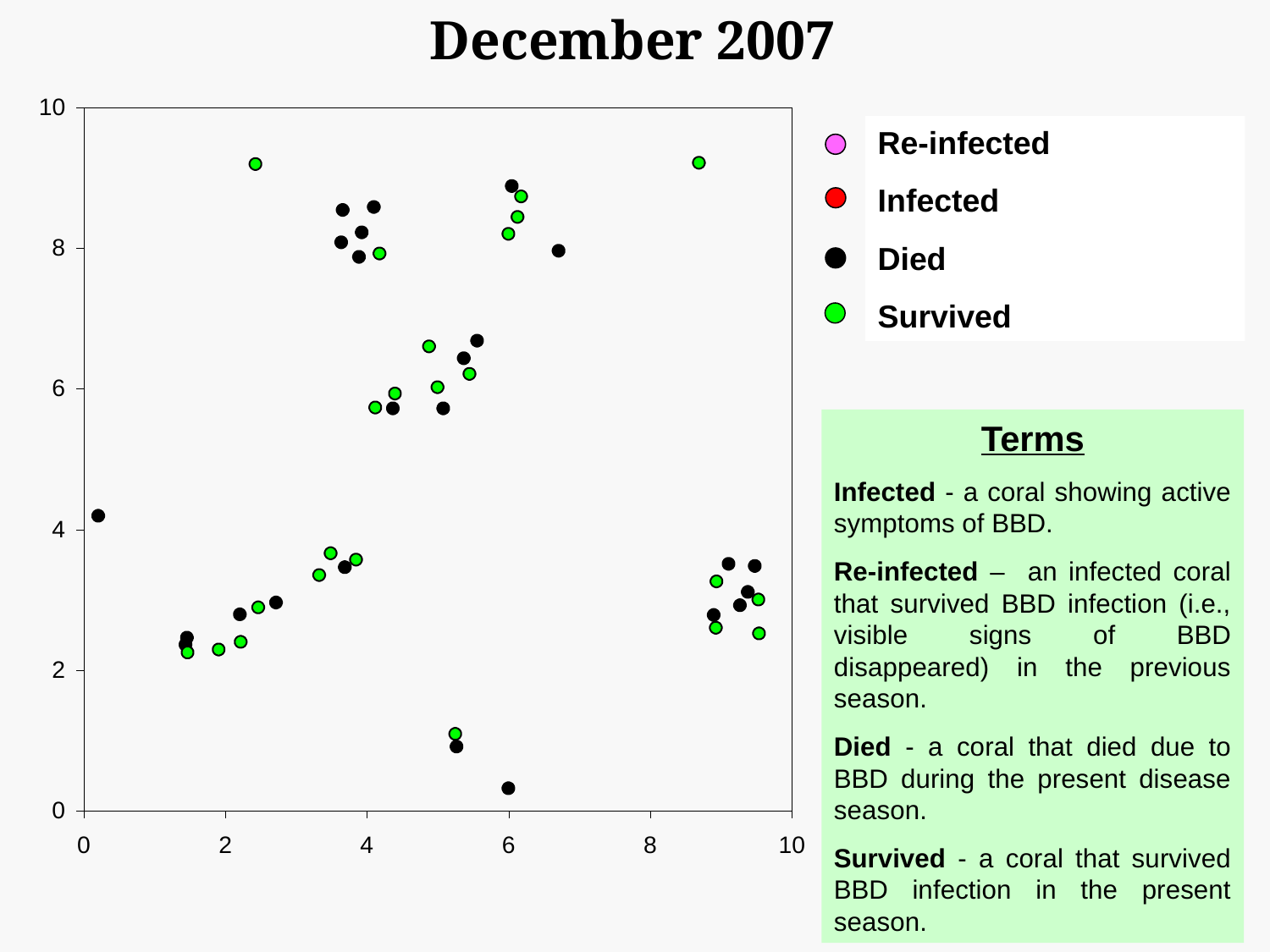

December 2007
Re-infected
Infected
Died
Survived
Terms
Infected - a coral showing active symptoms of BBD.
Re-infected – an infected coral that survived BBD infection (i.e., visible signs of BBD disappeared) in the previous season.
Died - a coral that died due to BBD during the present disease season.
Survived - a coral that survived BBD infection in the present season.
